# BUILDING FOUNDATIONAL COMPETENCIES IN OLDER ADULT MENTAL HEALTH: VIRTUAL AND IN-PERSON TRAINING

**DOI:** 10.1093/geroni/igad104.1873

**Published:** 2023-12-21

**Authors:** Erin Emery-Tiburcio, Laurin Mack, Siqi Wang, Susan Buehler

**Affiliations:** Rush University Medical Center, Chicago, Illinois, United States; Rush University Medical Center, Chicago, Illinois, United States; Rush University Medical Center, Chicago, Illinois, United States; Rush University Medical Center, Chicago, Illinois, United States

## Abstract

Designed to develop foundational knowledge competencies for generalist clinicians, this program was created by a team of geropsychologists, geriatric social workers, geriatric psychiatrists, and neuropsychologists, who reviewed competencies of multiple mental health disciplines, and identified 14 key areas of knowledge for clinicians. Content was created by Rush faculty, externally peer-reviewed, and developed into two versions. The 14-hour online version includes knowledge checks and national expert videos to provide multiple perspectives and voices of experience. Of the first 176 learners in the online version, 96.8% rated the overall program as excellent, with strong agreement (97.4 – 98.3%) that the training was applicable to practice, expectations were met, and learners had intention to apply the knowledge. All learners are required to achieve a minimum of 75% on knowledge post-test to complete each module. The three-day in-person program provided 25 diverse clinicians representing every HHS region with didactic content, interactive exercises, discussion, and a simulated patient experience to practice skills with live expert feedback. Participants demonstrated 14% improvement in knowledge (p<.01). Didactic lectures were rated 100% extremely or very helpful; 82% rated group learning exercises extremely helpful; 100% rated the simulation lab as extremely or very helpful. Balance between activities and match with learning style were rated good to very good. All participants rated the likelihood of recommending the program to a colleague as extremely or very likely. Evaluation of skill implementation is ongoing. Implications for expanding generalist competencies in older adult mental health will be discussed.

